# Depth-resolved Corneal Biomechanical Changes Measured Via Optical Coherence Elastography Following Corneal Crosslinking

**DOI:** 10.1167/tvst.10.5.7

**Published:** 2021-07-27

**Authors:** Tanner J. Ferguson, Srinidhi Singuri, Sanjai Jalaj, Matthew R. Ford, Vinicius S. De Stefano, Ibrahim Seven, William J. Dupps

**Affiliations:** 1 Cole Eye Institute, Cleveland Clinic, Cleveland, Ohio, USA; 2 Department of Ophthalmology, Cleveland Clinic Lerner College of Medicine of CWRU, Cleveland, Ohio, USA; 3 Department of Biomedical Engineering, Lerner Research Institute, Cleveland Clinic, Cleveland, Ohio, USA; 4 Department of Biomedical Engineering, Case Western Reserve University, Cleveland, Ohio, USA

**Keywords:** biomechanics, crosslinking, cornea, optical coherence elastography, OCT

## Abstract

**Purpose:**

To evaluate depth-resolved changes of corneal biomechanical properties in eyes with corneal ectasia after corneal crosslinking (CXL) using optical coherence elastography.

**Methods:**

In a prospective pilot series of eyes with corneal ectasia, a custom high-speed swept source optical coherence tomography system was used to image the cornea before and 3 months after CXL during a low-speed applanating deformation while monitoring applanation force. Cross-correlation was applied to track frame-by-frame two-dimensional optical coherence tomography speckle displacements, and the slope of force versus local axial displacement behavior during the deformation was used to produce a two-dimensional array of axial stiffness (*k*). These values were averaged for anterior (*k_a_*) and posterior (*k_p_*) stromal regions and expressed as a ratio (*k_a_/k_p_*) to assess depth-dependent differences in stiffness. CXL was performed according to the Dresden protocol with a system approved by the U.S. Food and Drug Administration.

**Results:**

Four eyes from four patients with keratoconus (*n* = 3) or post-LASIK ectasia (*n* = 1) underwent optical coherence elastography before and 3 months after CXL. The mean *k_a_/k_p_* was 1.03 ± 0.07 before CXL compared with 1.34 ± 0.17 after the CXL procedure. All four eyes demonstrated at least a 20% increase in the *k_a_/k_p_*_._

**Conclusions:**

Preferential stiffening of the anterior stroma with the standard CXL protocol was demonstrated with optical coherence elastography in live human subjects.

**Translational Relevance:**

Although ex vivo studies have demonstrated anterior stiffening effects after CXL using various destructive and nondestructive methods, this report presents the first evidence of such changes in serial live human measurements.

## Introduction

Keratoconus (KC), a form of corneal ectasia, is a leading cause of visual impairment worldwide.^1^ Recent studies^2^^,^^3^ investigating the prevalence and incidence of KC have indicated that the disease is considerably more prevalent than previously thought, likely owing to improved diagnostic and screening capabilities. A disease characterized by progressive thinning and distortion to the cornea, it remains a leading cause of corneal transplant in developing countries.^4^

Underlying abnormalities in the biomechanical properties of the cornea are postulated to be a major driving force in the development of corneal ectatic disorders such as KC and post-laser in situ keratomileusis (LASIK) ectasia.^5^^,^^6^ Although many of the screening and diagnostic criteria are based on morphologic characteristics such as thickness, curvature, and elevation,^7^^,^^8^ these observed morphologic changes are likely preceded by localized mechanical abnormalities. Numerous air puff–based approaches have been introduced to evaluate the biomechanical properties of the cornea including infrared reflectometry,^9^ Scheimpflug imaging^10^ and optical coherence tomography (OCT).^11^ Collectively, these imaging technologies have increased our understanding of corneal biomechanics, but all use a surface-based deformation assessment that limits their ability to assess local depth-dependent biomechanical properties and ultimately limits their clinical usefulness.

Optical coherence elastography (OCE), which was initially described in 1998^12^ and later developed for characterizing corneal biomechanics in 2011,^13^ uses swept-source OCT to acquire a series of images while mechanically applying an axial, applanation-like perturbation to the surface of the cornea. OCE allows for the characterization of spatial depth-dependent biomechanical properties and alterations within the cornea stroma. Recent reports by De Stefano et al.^14^^,^^15^ used OCE in live human studies of both normal and KC eyes and found that subjects with KC have a selective weakening of the anterior stroma, a finding that may prove to be a valuable marker for screening of KC and postsurgical ectasia risk.

The present study aims to investigate the depth-dependent biomechanical changes in subjects with corneal ectasia after corneal crosslinking (CXL) using OCE. The treatment of corneal ectasia has changed dramatically over the last decade with the advent of CXL, which was first described in 2003^16^ and was approved by the U.S. Food and Drug Administration in 2016. A procedure proven to stiffen the cornea, CXL is widely used for halting disease progression in KC and post-LASIK ectasia with favorable long-term results and stability.^17^ The use of OCE to assess depth-dependent changes in biomechanical properties after CXL may convey more direct effectiveness data for assessing and optimizing CXL treatment protocols and enhance our understanding of the underlying pathologic biomechanical changes driving corneal ectasia.

## Methods

This prospective study was approved by the Cleveland Clinic Institutional Review Board (IRB #13-213). All patients provided informed consent for research and for CXL treatment, and the study was conducted according to the Declaration of Helsinki. The study was registered at clinicaltrials.gov (NCT03030755).

A total of four eyes from four subjects previously diagnosed with corneal ectasia that underwent CXL were included in the study. All subjects were assessed by a fellowship-trained cornea and refractive surgeon (W.J.D.) with a confirmed diagnosis of corneal ectasia based on criteria listed in the American Academy of Ophthalmology Corneal Ectasia Preferred Practice Pattern guidelines.^18^ Of the four subjects, three had a diagnosis of KC and one had a diagnosis of post-LASIK ectasia. All subjects underwent a baseline ophthalmologic examination that included intraocular pressure measurement obtained with the Corvis ST (Oculus, Wetzlar, Germany), a noncontact tonometer, as well as corneal tomography with the Pentacam HR (Oculus). Data were collected before CXL (baseline) and at 3 months after the CXL procedure in the same eye to minimize the confounding effects of early corneal epithelial remodeling on tomographic results. At 3 months after CXL, corneal tomography difference maps were included to illustrate the change in corneal thickness and curvature after CXL. The CXL procedure was performed according to the Dresden protocol,^16^ the standard approach to CXL approved by the U.S. Food and Drug Administration, whereby the epithelium was removed and Riboflavin 0.146% drops (Photrexa Viscous, Avedro/Glaukos, San Clemente, CA) were instilled every 2 minutes for 30 minutes and for an additional 30 minutes with simultaneous exposure to 3 mW/cm^2^ UV-A irradiation (KXL system, Avedro).

The custom-built OCE system was described in detail in prior studies^13^^,^^14^ and is built around a 100 kHz swept source engine (HSL-20, Santec, Aichi, Japan). Scanning was performed at a rate of 100 k A-scans per second across a 5-mm horizontally oriented window with a 2-µm lateral oversampling.^14^ Before the imaging sequence, a drop of topical anesthetic was instilled into the study eye. Subject head fixation was accomplished using a bite plate with a disposable single-use cover (Ziploc, S.C. Johnson & Son, Racine, WI) for stability and motion artifact mitigation during the elastographic scan. Subject eye fixation was achieved by using a visual fixation target visualized through the OCT objective lens. The corneal perturbation interface – a flat, 3-mm-thick lens – was aligned with patent's optical axis and the interface was displaced in the axial direction through a total range of 2 mm over approximately 2 seconds. While the mechanical perturbation was delivered with a linear actuator (Parker Hannifin Corp, Cleveland, OH), 100 images (B Scan) were captured across the horizontal meridian while force sensors (LSB200, Futek, Irvine, CA) provided high-speed measurement of the force created by the contact of the interface with the cornea. For each subject, three compression sequences were performed to ensure that at least one usable deformation series was captured. Moreover, a three-dimensional volume was recorded for each eye for future use.

Displacement tracking for the OCT speckle pattern was conducted frame by frame on the raw OCT images akin to what was previously described in detail.^13^ A custom cross-correlation code was applied across all of the two-dimensional OCT images in the measurement sequence to generate the cumulative displacement data. The displacement data tracking was combined with the time-synced data from the force sensors to generate an estimate of axial stiffness, calculated as *k* = *f/d*, where *f* is the force generated from progressive contact between the interface and the anterior cornea and *d* is the local cumulative displacement of the cornea derived from the OCT speckle pattern tracking. Because the force/displacement relationship evolves along the compression sequence, *k* was defined as the slope of a line fit to the force/displacement data.^14^ To distinguish the anterior/posterior regions of the corneal stroma, two 1.6-mm (wide) × 150 µm (axial depth) regions were defined and *k* values were calculated and averaged across each region to generate an anterior (*k_a_*) and posterior (*k_p_*) axial stiffness measure. These two defined regions were separated axially by approximately 120 µm. These values were expressed as a ratio (*k_a_/k_p_*) to assess depth-dependent differences in axial stiffness. A ratio (*k_a_/k_p_*) value of 1 would indicate equivalence between the axial stiffness in the anterior and posterior regions, whereas values of more than 1 would indicate a stiffer anterior stroma and values or less than 1 indicate a stiffer posterior stroma.

To assess the force/displacement relationship as it evolves across the compression sequence, graphs were generated for each subject. It is also important to note that the relationship evolves temporally across the sequence, which allows for a comparison of displacement values between the anterior and posterior regions of the cornea for any given point in time or force (*f* on the *y*-axis) in the sequence. These graphs allow assessment of the depth-dependent difference in the loading behavior of the cornea.

The three replicate OCE measurement sequences were obtained for each eye by the same observer in a span of less than 15 minutes. Only one sequence for each eye was sent for processing of *k_a_*/*k_p_* and was selected on the basis of motion artifact and relative absence of data gaps in the perturbation sequence. The *k_a_/k_p_* ratio was calculated at baseline before the CXL procedure and again after the CXL procedure at 3 months to assess the changes in axial stiffness after CXL.

## Results

**Figure 1.**
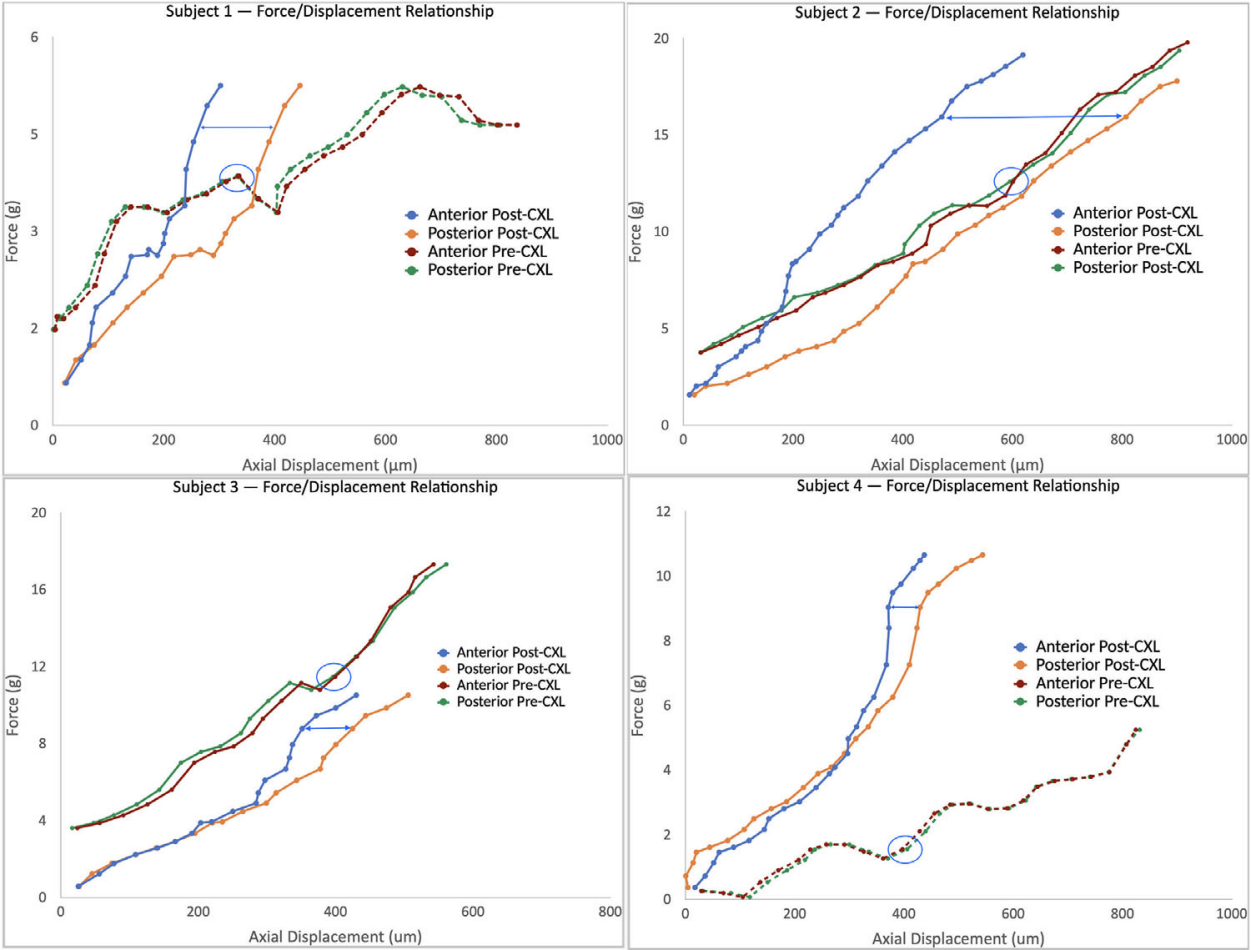
Serial same eye comparison plots of applanation force (in grams) versus axial stromal displacement (in micrometers) of anterior and posterior corneal regions of interest for 4 eyes before and after corneal CXL. Anterior and posterior data are obtained simultaneously with OCE along a two-dimensional scan along the horizontal meridian. The difference in deformability under the same surface load can be seen by comparing the *x*-axis (displacement) position for the 2 regions along any *y*-axis (force) value (*blue horizontal lines* in post-CXL and *blue circles* in pre-CXL, where the differences for the later are too small to illustrate with a line). For example, in subject 1, the anterior stroma (*burgundy*) shows slightly greater displacement (higher deformability) than the posterior stroma (*green*) across most of the deformation curve. After CXL, the anterior stroma (*blue*) is far less deformed than the posterior stroma (*orange*), indicating a marked stiffening of the anterior stroma. Slopes are fit to each curve to characterize the overall force/displacement relationship for each region to yield a measure of axial stiffness (see text). The slope term is insensitive to slight differences in initial force/preload between the pre-CXL and post-CXL comparison, as can be seen in subject 3 (*lower left*), where there is a notable increase in the slope (stiffness) of the post-CXL anterior stroma in the latter portion of the perturbation sequence.

All four eyes of four patients were imaged at baseline then again 3 months after the CXL procedure. Table 1 demonstrates the demographic and tomographic characteristics at baseline and at 3 months after CXL. Graphs depicting the force/displacement relationship pre- and post-CXL for each subject are depicted in Figure 1. The displacement data tracking is time synced, which permits comparison of the displacement of the anterior and posterior regions of the cornea at any given force in the applanation sequence. There are distinct patterns evident on the force/displacement across the 4 subjects before and after CXL. For all four subjects, before CXL, there is minimal difference in the displacement distance (micrometers) between the anterior and posterior regions of the cornea. In addition, for the pre-CXL measurements, there is greater displacement associated with the anterior region compared with posterior at many segments across the loading curve. After CXL, in all four subjects, in contrast, there is a decrease in displacement in the anterior region compared with the posterior region of the cornea. This relationship – decreased displacement anteriorly versus posteriorly – is clearly evident at higher magnitudes of force (*g*) along the loading curve in all four subjects after CXL.

**Table 1.** Baseline and Follow-up Characteristics of Study Participants. Post-CXL Measurements Obtained From Scheimpflug Tomography Images Obtained 3 Months Post-CXL

						Pre-CXL	Pre-CXL	Pre-CXL	Post-CXL	Post-CXL	Post-CXL
Subject	Age (Years)	Diagnosis	Sex (M/F)	SE MRx (D)	IOP (mm Hg)	K_m_ (D)	K_max_ (D)	TPCT (µm)	K_m_ (D)	K_max_ (D)	TPCT (µm)
01	23	KCN	F	+1.875	9.0	51.8	69.5	408	51.8	68.9	415
02	26	KCN	M	+2.75	15.0	52.6	63.0	398	51.2	61.5	391
03	21	KCN	M	−0.625	16.0	43.5	51.2	495	43.1	51.6	481
04	49	Post-LASIK ectasia	M	−1.75	9.5	43.6	64.3	390	49.4	70.3	373

MRx, manifest refraction; IOP, intraocular pressure; K_m_, mean keratometry; K_max_, maximum keratometry value; TPCT, thinnest point of corneal thickness (from Scheimpflug tomography).

**Figure 2.**
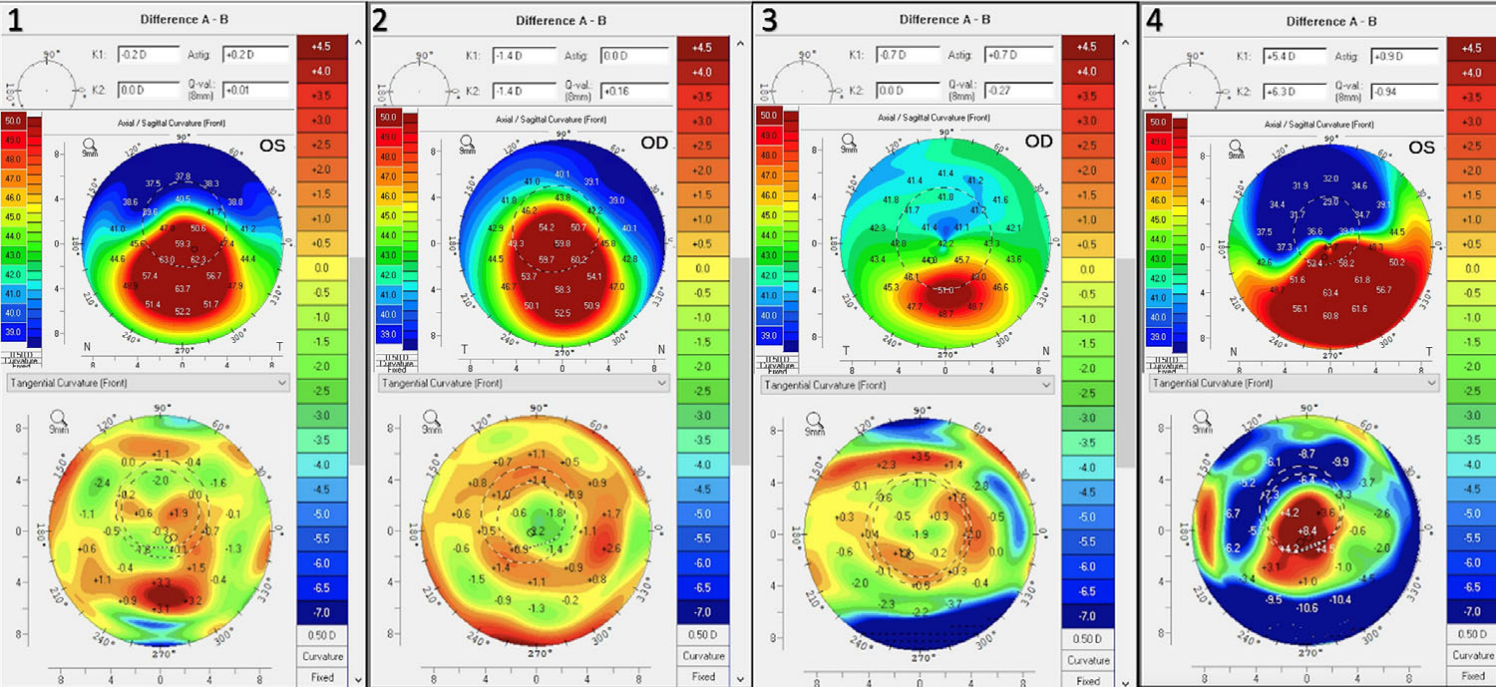
Preoperative axial topography and 3-month difference map for each study eye.

Scheimpflug tomography difference maps are demonstrated in Figure 2. Subjects two through four all demonstrated a decrease in the TPCT (thinnest point of corneal thickness) value. Subjects one, two, and three demonstrated a decrease in central keratometry values consistent with flattening. Subject four, a post-LASIK ectasia patient, demonstrated central steepening that corresponded to an increase in the maximum keratometry value by more than 6 diopters.

To assess the changes after CXL from a quantitative perspective, *k* ratios before and after linking were compared. As discussed elsewhere in this article, the *k_a_/k_p_* values are derived from the slope of a linear fit to the force/displacement data over the entire compression sequence. At baseline, the *k_a_/k_p_* was 1.03 ± 0.07 with all four eyes measuring *k_a_*/*k_p_* values of less than 1.1 (range, 0.93–1.09). After the CXL procedure, the mean *k_a_*/*k_p_* value was 1.34 ± 0.17, a 34% (0.31) mean increase from baseline. All four subjects demonstrated a percentage increase in *k_a_*/*k_p_* of more than 20% from baseline and the post-CXL *k_a_*/*k_p_* values ranged from 1.06 to 1.58. The values for each subject are demonstrated in Table 2.

**Table 2.** Anterior to Posterior Stromal Stiffness Ratio K_a_/K_p_ for Each Subject Before and 3 Months After Epithelium-off CXL

	Age		K_a_/K_p_	K_a_/K_p_
Subject	Years	Eye	Pre-CXL	Post-CXL
01	23	Left	0.934	1.579
02	26	Right	1.044	1.235
03	21	Right	1.051	1.205
04	49	Left	1.087	1.349

## Discussion

This study describes the use of OCE to characterize serial depth-dependent biomechanical changes owing to CXL, a procedure that has been shown with destructive bulk testing methods to increase corneal tensile strength.^16^ Anterior stromal stiffening after CXL has been demonstrated nondestructively in prior ex vivo work using Brillouin microscopy in porcine eyes.^19^^,^^20^ To our knowledge, only one prior study has presented in vivo human data showing depth-dependent biomechanical property changes after CXL in live human eyes,^21^ and that work involved a noncontact OCT-based phase decorrelation analysis in two eyes that had undergone prior CXL but did not assess serial changes. This study is the first to provide serial evidence of anterior stromal stiffening in live human subjects. A prior study by De Stefano et al.^15^ using OCE reported a disruption of the normal elasticity gradient pattern with selective weakening of the anterior stroma in human eyes with KC relative to normal eyes. Collectively, the OCE evidence to date have important implications for understanding the biomechanical benefits of CXL by demonstrating a selective stiffening of the anterior stroma in the same region shown to be preferentially weakened in eyes with KC. These results provide direct biomechanical evidence of anterior stiffening in CXL that cannot be ascertained with current anterior corneal deformation analysis methods.

The *k_a_*/*k_p_* results after CXL quantitatively support the notion that CXL, using the protocol described in this report, confers a selective stiffening in the anterior stroma with a more than 20% increase in the anterior–posterior stiffness gradient occurring across all four subjects. The force/displacement graphs in Figure 1 offer additional insight into the biomechanical changes after CXL and highlight the region-specific changes. Before CXL, as shown in the graphs, there is a minimal difference across all four subjects in the displacement along the loading curve between the anterior and posterior region of the cornea, indicating similar degrees of stiffness. The post-CXL plots depict a transformation in the relationship between the anterior/posterior cornea with a decreased displacement anteriorly versus posteriorly; this feature is most evident at higher levels of force along the loading curve for all four subjects, but was captured by the slope-based k metrics in every case. The topographic difference maps at 3 months show a variety of shape responses to CXL, most with some localized flattening in the cone region, but with the one eye affected by post-LASIK ectasia demonstrating central steepening despite evidence of a significant increase in stiffness (Fig. 1). In this subject (subject four), subsequent follow-up at 2 years after CXL demonstrated no further topographic progression after 3 months. These early topographic outcomes, which can be confounded by post-CXL epithelial changes and other wound healing effects, highlight the underlying limitations of using corneal curvature responses as a proxy for a stiffening effect and CXL efficacy.

The in vivo data obtained in this early translational series offer several advantages. In vivo measurements preserve the integrity of the corneal anatomy and hydration levels, both of which are notably altered and difficult to maintain when removed from their natural boundary conditions in ex vivo experiments. Numerous prior studies have demonstrated the challenges in accurately assessing biomechanical properties of the cornea with study conditions that affect hydration levels.^22^^–^^25^ Using the 2-second measurement applied to live subjects in this present study, the risk of altered hydration is minimized and regional corneal displacements more accurately reflect the cornea's behavior with its native boundary conditions.

This study has limitations, most notably the small sample size. Future, larger scale studies will be important for confirming the findings observed in this study with regard to changes after CXL. Although contralateral eye measurements might have been useful as a short-term control for nonintervention, three of the four subjects had a prior contralateral eye CXL when enrolled. The statistical design used a serial same eye control scheme to emphasize eye-specific changes and to avoid the potential confounding effects of interocular asymmetry and intersubject variability in KC. The current technique for obtaining OCE has limitations, including the theoretical impact of intraocular pressure on measurements. However, because anterior and posterior portions of the cornea are subject to the same forces with each measurement, it is unlikely the anterior and posterior deformation relationships are strongly influenced by the intraocular pressure. This concern was addressed at length in a prior report with supporting data and computational modeling sensitivity analyses.^14^ Additionally, thus far, clinical OCE analysis has emphasized axial displacement data in a two-dimensional plane across the corneal center. As demonstrated in prior OCE work,^25^ anterior lateral displacement in the cornea is also reduced after CXL. Simultaneous capture of lateral displacement data is integral to the cross-correlation analysis but interpretation is currently hampered by patient motion. Because lateral and out-of-plane displacements will likely complement axial displacement data, efforts are underway to expand OCE to account for three vector component displacements across three-dimensional volumes to maximize displacement tracking fidelity and support enhanced regional sampling and property sensitivity.

## Conclusions

This study provides biomechanical evidence of a selective increase in anterior stromal stiffness properties after CXL in human eyes with corneal ectasia. Clinical OCE could be useful for assessing the relative stiffening effect of different CXL protocols, evaluating the biomechanical consequences of different refractive surgeries such as LASIK, PRK and small incision lenticule extraction, and improving the safety and predictability these procedures based on patient-specific measurements of spatial biomechanical property profiles.
